# COVID-19 Presenting as Encephalitis and Myopericarditis: A Report of a Rare Case

**DOI:** 10.7759/cureus.62056

**Published:** 2024-06-10

**Authors:** Nikolaos Kakaletsis, Ioannis Alevroudis, Serafeim-Chrysovalantis Kotoulas, Vasiliki Dourliou, Maria Stougianni, Eleni Massa, Eleni Mouloudi

**Affiliations:** 1 Internal Medicine Unit, Ippokrateio General Hospital, Aristotle University of Thessaloniki, Thessaloniki, GRC; 2 Cardiology Unit, Ippokrateio General Hospital, Aristotle University of Thessaloniki, Thessaloniki, GRC; 3 Adult Intensive Care Unit, Ippokrateio General Hospital, Thessaloniki, GRC

**Keywords:** case-report, cardiac tamponade, myocarditis, pericarditis, encephalitis, coma, covid-19

## Abstract

COVID-19 might present with a wide range of clinical manifestations, from mild respiratory distress to severe multi-organ dysfunction. We present a unique case of complex COVID-19 presentation in a 45-year-old female who initially developed general symptoms such as fever, cough, headache, and weakness, which escalated to coma, requiring intubation and ICU admission. A brain MRI revealed lesions compatible with encephalitis, the cause of which remained unexplained after an in-depth clinical, laboratory, and imaging investigation. While in the ICU, the patient also developed cardiac tamponade, requiring pericardiocentesis, and atypical electrocardiographic changes. After treatment with steroids, her condition improved, and the patient was extubated and transferred to the ward. Upon checkup, cardiac MRI revealed fibrous tissue in the inferior cardiac wall and the adjacent intraventricular septum. In the absence of an alternative diagnosis, it might be important to consider the central nervous system and cardiac involvement in patients with COVID-19.

## Introduction

COVID-19 usually presents as a respiratory disease with a wide range of clinical manifestations, spanning from asymptomatic cases to severe acute respiratory distress syndrome [1]. Recent studies revealed that COVID-19 might also impact the nervous system [2], with stroke and anosmia being some of its most common neurological complications [3]. Neurological manifestations of COVID-19 can be mild, such as headache, anosmia, and dizziness, or severe, such as encephalitis, encephalomyelitis, and stroke [4,5]. In addition to that, the prevalence of COVID-19-related cardiac diseases is relatively high, and pericarditis-myocarditis is a well-known extrapulmonary manifestation [6,7]. However, pericardial effusion leading to cardiac tamponade is less prevalent and may require urgent intervention to prevent death [8].

## Case presentation

We present the case of a previously healthy 45-year-old female who presented to the emergency department with a three-day history of high fever, cough, persistent headache, and weakness, indicative of COVID-19. She had completed her vaccination for COVID-19 with three doses of the mRNA vaccine 10 months earlier. Within a few hours, she developed lethargy and deteriorated into a comatose state (Glasgow Coma Scale score: 6/15). As a result, she was intubated and transferred to the ICU.

Clinical course

On admission, COVID-19 was confirmed by an RT-PCR test (predominant phase of SARS-CoV-2 Omicron sublineage ΒΑ.2 και ΒΑ.5 in Greece) [9].

**Figure 1:**
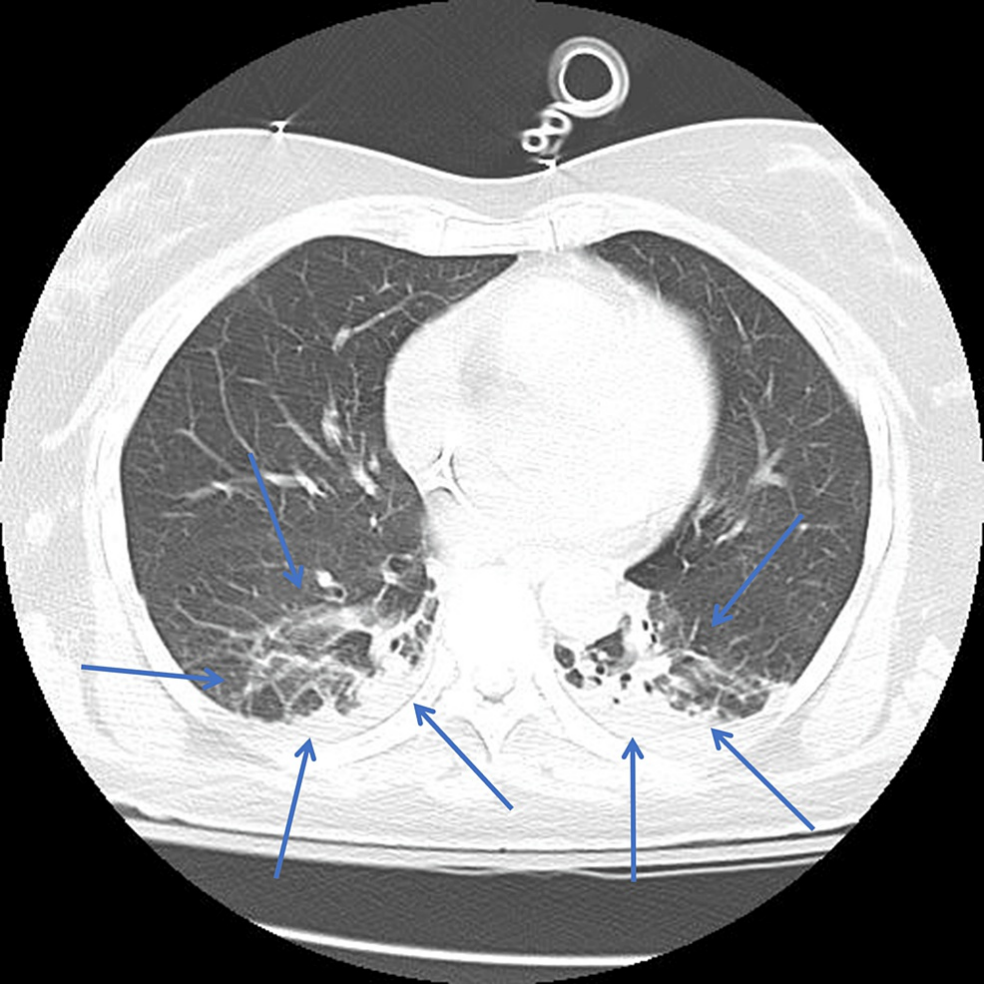
CTPA showing focal infiltrates in the lower lung lobes (blue arrows) CTPA, CT pulmonary angiogram

Upon initial evaluation, she was sedated on mechanical ventilation, with normal vital signs and an ECG showing no abnormalities. A chest X-ray revealed focal infiltrates in the lower lung lobes, while a CT pulmonary angiogram confirmed these findings and ruled out pulmonary embolism (Figure 1).

**Figure 2:**
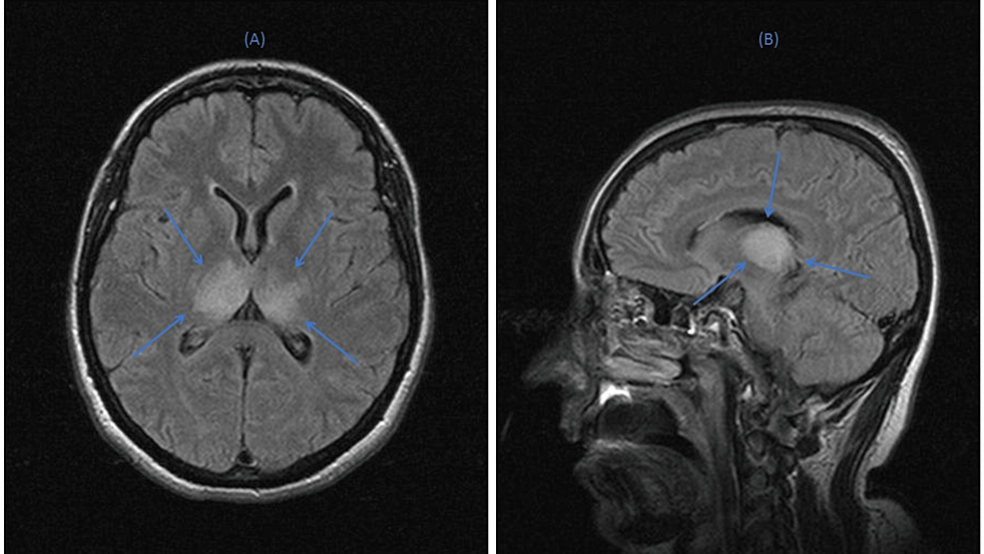
Brain MRI images in (A) axial and (B) sagittal planes, illustrating bilateral thalamic infiltrates (blue arrows)

Brain CT was unremarkable; however, brain MRI the next day revealed infiltrates in both thalami on T2/fluid-attenuated inversion recovery (FLAIR) images, with no significant contrast agent enhancement after gadolinium injection (Figure 2), suggesting either ischemic stroke or encephalitis.

The initial complete blood count was unremarkable, with a white blood cell count of 8.10 Κ/μL (neutrophils: 6.6 Κ/μL and lymphocytes: 1.2 Κ/μL), a hematocrit of 34.7%, and a platelet count of 172,000 μL. Biochemical analyses showed a CRP at 8.5 mg/dL (normal range: <6 mg/dL), procalcitonin at 0.02 ng/mL, erythrocyte sedimentation rate at 12 mm/hour, lactate dehydrogenase at 273 IU/L (normal range: <248 IU/L), creatine phosphokinase at 79 IU/L, ferritin at 35.9 g/mL, D-dimer at 1,037 μg/L (normal range: <500 μg/L), and elevated levels of cardiac troponin I high sensitivity at 51.9 pg/mL (normal range: <11.6 pg/mL) (Table 1).

**Table 1:** Blood and biochemical analysis results ALP, alkaline phosphatase; ALT, alanine aminotransferase; AST, aspartate aminotransferase; CPK, creatine phosphokinase; ESR, erythrocyte sedimentation rate; GGT, gamma-glutamyl transferase; LDH, lactate dehydrogenase

Laboratory tests	Reference range	Values
WBC (K/μL)	4.5-10.5	8.1
Neutrophil (%)	40-75	82.3
Hemoglobin (g/dL)	11.5-14.5	11.5
Hematocrit (%)	35-47	34.7
Platelet count (K/μL)	150-400	172
AST (U/L)	10-31	30
ALT (U/L)	10-34	44
CRP (mg/dL)	<6	8.5
Procalcitonin (ng/mL)	<0.5	0.02
Glucose (mg/dL)	75-100	117
Urea (mg/dL)	10-43	19
Creatinine (mg/dL)	0.66-1.10	0.6
Ca (mg/dL)	8.8-10.6	7.4
P (mg/dL)	2.5-4.5	3
K^+^ (mmol/L)	3.5-5.1	4.3
Νa^+^ (mmol/L)	136-145	136
Total protein (g/dL)	6.6-8.3	5.8
Albumin (g/dL)	3.5-5.2	3.6
ΑLP (U/L)	30-120	63
Total bilirubin (mg/dL)	0.3-1.2	0.44
Mg^++^ (mg/dL)	1.6-2.3	2.4
LDH (U/L)	<248	273
CPK (U/L)	<145	79
GGT (U/L)	<38	25
Troponin Ι high sensitivity (pg/mL)	<11.6	51.9
Ferritin (ng/mL)	11.0-306.8	35.9
D-dimer (ng/ml)	<500	1,037
ESR (mm/hr)	<19	12

Serology testing for hepatitis B virus, hepatitis C virus, HIV, varicella-zoster virus, parvovirus B19, cytomegalovirus, Epstein-Barr virus (EBV), Rubella virus, *Toxoplasma gondii*, measles virus, and West Nile virus were negative, while blood PCR for EBV and adenovirus also came back negative. Her blood, urine, and bronchoalveolar lavage cultures were also negative, as were her blood, urine, and gastric lavage toxicological exams.

A lumbar puncture was also performed, and CSF analyses were negative for infection (Table 2). Only CSF protein levels were found to increase, suggesting inflammation. Further investigation of serum for antibodies targeting intracellular and neuronal cell surface antigens was also negative. Other immune-mediated neurological disorders, including autoimmune encephalitis, stiff-person syndrome, progressive encephalomyelitis with rigidity and myoclonus syndrome, and paraneoplastic neurological syndromes, were also ruled out through autoantibody detection using the indirect fluorescent antibody assay and immunoblotting in serum. In particular, IgG autoantibodies for antigens of the central nervous system such as NMDA-R, AMPA-R1/2, GABAB-R, LGI1, dopamine-R2, DPPX, GluRδ2, IgLON5, mGluR1, mGluR5, CASPR2, glycine receptor, neurochondrin, ITPR1, ΑΝΝΑ-3, AGNA, CARPVIIΙ, PCA-2, Rho-GTPase activating protein 26, Homer 3, amphiphysin, CV2/CRMP5, PNMA2 (Ma2/Ta), Ri-ΑΝΝΑ-2, Yo/PCA-1, Hu/ΑΝΝΑ-1, recoverin, SOX1, Zic4, and Tr(DNER) (both in serum and in CSF, where applicable) were all negative.

**Table 2:** CSF analysis CMV, cytomegalovirus; LDH, lactate dehydrogenase; WNV, West Nile virus

CSF	Values
Color	Colorless (reference range: colorless)
WBC count (mm^3^)	0 (reference range: 0-5)
Red blood cells (mm^3^)	0 (reference range: 0)
Glucose (mg/dL)	68 (reference range: 40-70)
Protein (mg/dL)	176 (reference range: 15-45)
LDH (IU/L)	20 (reference range: <20.0)
Gram stain	Negative
*Escherichia coli* K1 PCR	Negative
*Haemophilus influenzae *PCR	Negative
*Listeria monocytogenes *PCR	Negative
*Neisseria meningitidis* PCR	Negative
*Streptococcus agalactiae *PCR	Negative
*Streptococcus pneumoniae* PCR	Negative
CMV PCR	Negative
Enterovirus PCR	Negative
Herpes simplex virus 1-2 PCR	Negative
Human herpesvirus 6 PCR	Negative
Human parechovirus PCR	Negative
Varicella zoster virus PCR	Negative
*Cryptococcus neoformans/gatti* PCR	Negative
Culture	Negative
SARS-CoV-2 RT-PCR	Negative
Anti-WNV IgM	Negative
Anti-NMDA-R IgG	Negative

In the absence of an alternative diagnosis, the patient was diagnosed with COVID-19-related encephalitis and was initiated on treatment with remdesivir (200 mg*1 on the first day and 100 mg*1 for four more days) and immune modulation therapy with methylprednisolone (1 g/day for five days, followed by 0.5 mg/kg/day for five more days). She also received antiepileptic treatment with levetiracetam (1 g/day), while, even though a diagnosis of ischemic stroke seemed rather unlikely, due to the distribution of the lesions on brain MRI, she also received treatment with acetylsalicylic acid.

Cardiac involvement

**Figure 3:**
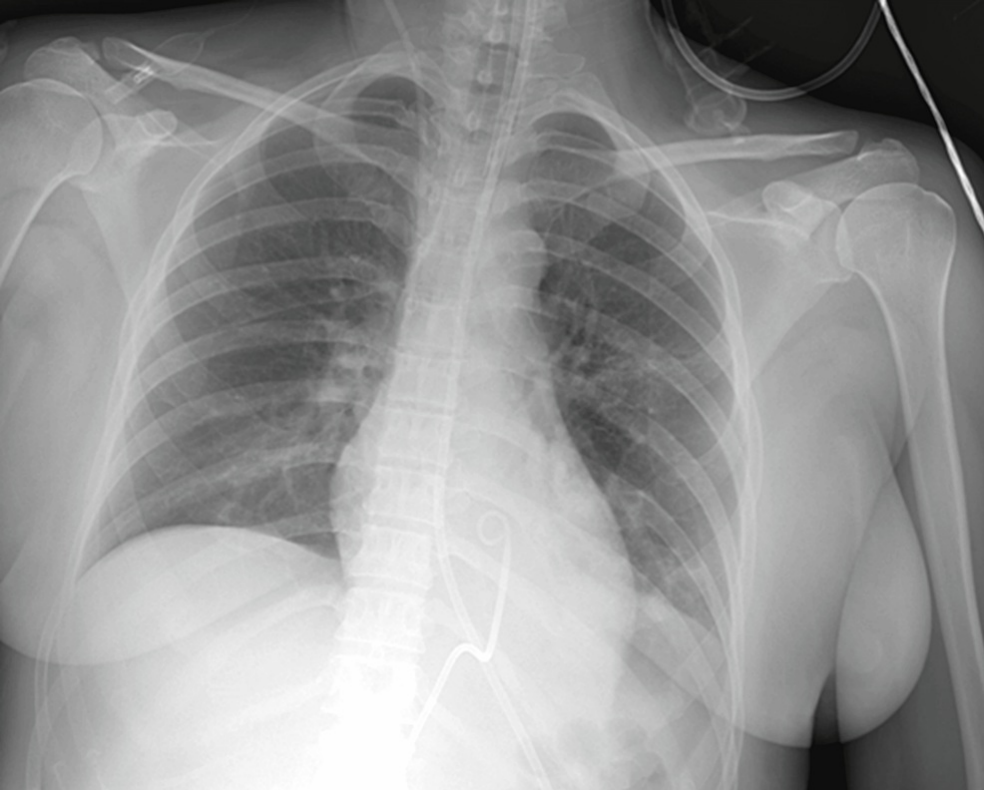
Chest X-ray showing a normal cardiac silhouette with the catheter positioned in the pericardial cavity just before its removal

The second day after ICU admission, the patient developed severe hemodynamic instability, which escalated within a few minutes, presenting sinus tachycardia with electrical alternans, pulsus paradoxus, and an extreme increase in the need for vasoconstrictive support. Bedside heart ultrasound revealed a pericardial effusion of approximately 15 mm with right atrium tamponade. Unfortunately, due to the urgent conditions during the pandemic, it was not possible to record the echocardiography images. Echo-guided pericardiocentesis was performed, and approximately 200 ml of pericardial fluid (PF) was removed instantly, with no further PF drainage the next three days that the catheter remained in the patient’s pericardial cavity (Figure 3) since colchicine was added to her treatment.

After PF drainage, the patient was again stabilized hemodynamically. Table 3 shows the results from PF analysis, while a subsequent ECG revealed a non-preexisting T-wave inversion in V1-V6 leads.

**Table 3:** PF analysis CMV, cytomegalovirus; EBV, Epstein-Barr virus; LDH, lactate dehydrogenase; PF, pericardial fluid

PF	Values
Appearance	Slightly cloudy (reference range: clear)
Color	Serosanguinous (reference range: straw colored)
WBC count (mm^3^)	5 cells/mm^3^
Red blood cells (mm^3^)	Plenty (reference range: none)
Protein (g/dL)	0.192 (reference range: <3 g/dL)
LDH (IU/L)	17 IU/L
Gram stain	Negative
Bacterial/fungal culture	Negative
*Haemophilus influenzae *PCR	Negative
*Listeria monocytogenes *PCR	Negative
*Neisseria meningitidis* PCR	Negative
*Streptococcus agalactiae* PCR	Νegative
*Streptococcus pneumoniae* PCR	Negative
CMV PCR	Negative
Human enterovirus PCR	Negative
Herpes simplex virus 1-2 PCR	Negative
Human herpesvirus 6-7-8 PCR	Negative
Human parechovirus PCR	Negative
Varicella zoster virus PCR	Negative
*Cryptococcus neoformans/gatti *PCR	Negative
Mumps virus PCR	Negative
Measles virus PCR	Negative
EBV PCR	Negative
*Staphylococcus aureus* PCR	Negative
*Borrelia burgdorferi/miyamotoi *PCR	Negative

Further PF cytological analysis came back negative for malignancy, while cytofluorometric analysis revealed lymphocytes at approximately 66.8%. RT-PCR testing for COVID-19 in PF was not available; however, in the absence of malignancy, chest trauma, and coagulopathy, the pericardial effusion was attributed to the patient’s COVID-19 infection.

Recovery: continued care

Following methylprednisolone pulses, sedation was withdrawn, and the patient’s neurological condition gradually improved. On the 12th day after ICU admission, she was successfully extubated. Apart from a mild short-term memory impairment, her neurological status was otherwise normal. Two days later, she was discharged from the ICU while receiving oxygen therapy at 4 liters per minute via nasal cannula. The patient continued her treatment with colchicine for the rest of her hospitalization. Repeated heart ultrasounds prior to discharge demonstrated stable cardiac function without pericardial effusion.

After discharge, her 24-hour Holter monitor revealed episodes of supraventricular tachycardia and isolated ventricular ectopic beats, while her T-wave inversion in precardiac leads resolved. The patient did not suffer any syncope or loss of consciousness episodes; her heart ultrasound remained normal, while her neurological status reverted to that before hospitalization. Her cardiac MRI, however, revealed subendocardial accumulation of contrast solution in the inferior wall of the left ventricle and, to a smaller extent, in the adjacent interventricular septum after the delayed myocardial enhancement.

Diagnostic challenges

Despite extensive diagnostic efforts, the exact cause of the patient’s coma and cardiac tamponade remained unclear, since repeated brain imaging and laboratory testing have not provided a definite cause. The most probable explanation for this multifaceted clinical presentation is a COVID-19 infection affecting both the central nervous system and the heart.

## Discussion

The pathophysiological mechanism of acute encephalitis in COVID-19 is not well defined. Several mechanisms could explain neurological manifestations associated with COVID-19, including direct neural invasion, para- or post-infectious immunological disorder, potential neurotoxicity of therapeutic agents, and toxic/metabolic encephalopathy, especially in the context of the high burden of proinflammatory cytokines that characterizes severe COVID-19 [10-14].

The two main hypotheses are a direct cytopathic effect of the infection on the brain tissue (the angiotensin-converting enzyme 2 receptors that the virus uses for attachment, margination, and internalization in the lungs are also expressed in the central nervous system; viral antigens have been detected in CSF and brain samples) and an autoimmune/immune-mediated cause (the general hyperinflammatory state releases cytokines and chemokines that impair the blood-brain barrier permeability and activate neuro-inflammatory cascades) [13-16]. In our patient, we presume the causative factor was immune mediated, given the notable response to intravenous steroids.

The diagnosis of acute encephalitis is based on clinical presentation, CSF analysis, and brain imaging. However, there are no diagnostic criteria for COVID-19-induced encephalitis, as the definitive diagnosis of viral encephalitis is based on viral isolation from CSF. Viral isolation is difficult due to the transient dissemination of COVID-19 and its low CSF titer [17]. Consequently, the absence of COVID-19 in the CSF sample does not rule out the diagnosis [13,14]. In our case, the PCR assay for COVID-19 was negative, not allowing a definitive diagnosis of viral encephalitis. A case series comprising CSF analysis data from 32 patients with SARS-CoV-2-associated encephalitis/meningitis reported that the PCR assay for COVID-19 in the CSF was negative in 87.5% of the cases [17], while in another case series, involving 40 patients, it was negative in 90% of the cases [16]. Brain CT and MRI are usually performed to identify the classic findings of encephalitis. COVID-19-induced encephalopathy has shown a broad spectrum of MRI findings such as leptomeningeal enhancement, white matter microhemorrhages, FLAIR signals, and ischemic strokes [18].

Management protocols for COVID-19-induced encephalitis include primarily supportive therapy [3,17]. Modes of management that have yielded positive patient outcomes include corticosteroids [19], intravenous immunoglobulin [20], plasmapheresis [21], and monoclonal antibodies such as rituximab [22]. Nevertheless, it is important to interpret the initial success of these treatment modalities with caution, given the lack of large-scale randomized control trials assessing their efficacy. Further research is necessary in this domain to ascertain their suitability as treatment modalities for COVID-19-induced encephalitis.

Regarding cardiovascular manifestations that have been reported secondary to SARS-CoV-2 infection, they are very heterogeneous, ranging from mild symptoms, such as chest pain, to severe ones, such as myocardial injury, acute heart failure, and arrhythmias [23]. COVID-19-related pericardial involvement is rare, and as a result, very few cases of cardiac tamponade have been reported in the literature. However, cardiac tamponade as a life-threatening complication of myopericarditis in COVID-19 is an important differential to consider in a hemodynamically deteriorating patient with COVID-19 [24].

There are no specific laboratory parameters to differentiate COVID-19-related pericardial effusion from other etiologies. Until RT-PCR testing for COVID-19 on PF becomes more widely available, a complete biochemical, bacteriological, and cytological PF analysis to rule out other causes of pericardial effusion is suggested.

Pericardial effusion associated with viral infections can exhibit various appearances depending on the specific viral etiology and the stage of the infection, such as the following: (1) Transparent or straw-colored fluid (in the early stages of viral pericarditis, before significant inflammation or cellular infiltration occurs, the PF may appear transparent or straw-colored, resembling a transudative effusion). (2) Serous or serohematic fluid: as the viral infection progresses and inflammation of the pericardium ensues, PF may become serous or serohematic, with a slight pink or yellowish tinge. This appearance reflects increased vascular permeability and leakage of plasma proteins and red blood cells into the pericardial cavity. (3) Cloudy or turbid fluid: in more severe cases or in the presence of secondary bacterial superinfection, PF may become cloudy or turbid due to the presence of inflammatory cells, cellular debris, and microorganisms. This appearance suggests an exudative effusion with significant inflammation and infectious involvement. (4) Bloody or hemorrhagic fluid: pericardial effusion associated with certain viral infections, particularly those causing severe myocardial involvement or hemorrhagic manifestations, may appear bloody or hemorrhagic. Hemorrhagic pericardial effusion is a concerning finding and may indicate a more severe course of the viral infection or complications such as myocardial rupture. (5) Fibrinous or fibrinopurulent fluid: in cases of prolonged or severe viral pericarditis, PF may become fibrinous or fibrinopurulent, with the formation of fibrin strands or loculations within the effusion. This appearance suggests ongoing inflammation and fibrin deposition, which may lead to the development of constrictive pericarditis if not treated adequately. Thus, the clarity and consistency of PF may vary depending on the specific viral etiology, host immune response, and presence of a concomitant bacterial or fungal infection. Early viral pericardial effusions may be relatively clear and serous, while later stages or severe cases may exhibit cloudiness, turbidity, or hemorrhage.

It is important to note that the appearance of pericardial effusion in viral infections is not pathognomonic and may overlap with other causes of pericardial inflammation. A comprehensive evaluation, including clinical history, physical examination, laboratory tests, and imaging studies, is necessary to establish the diagnosis and guide appropriate management. In suspected cases of viral pericarditis, viral serology, PCR testing, and culture studies may be performed on PF to identify the causing virus and guide treatment decisions.

Current data, which are very limited, suggest that the pericardial effusion seen in COVID-19 is sterile, suggesting an inflammatory response rather than a pericardial infection. Therefore, the fluid appears to be exudative and free of viruses. In our case, the fluid was also sterile but transudative, probably due to the fulminant invasion and the early stage of the disease. Additionally, the degree of the pericardial inflammatory response is not related to the severity of myocarditis; consequently, tamponade can be present in the absence of severe or fulminant myocarditis.

A meta-analysis suggests that approximately 5% of patients with COVID-19 who undergo chest CT in a clinical context present a detectable pericardial effusion [25]. An unexplained hemodynamic failure or increased cardiac biomarkers should be alarming for myopericarditis and lead to transthoracic echocardiography and an ECG. An analysis of the aspirated fluid should be performed, which, in the majority of cases, is expected to be negative for COVID-19.

As far as the pathogenesis of COVID-19 myopericarditis is concerned, it is not yet resolved. There are two possible mechanisms. The first one involves the direct binding of the COVID-19 protein to human angiotensin-converting enzyme 2 [26], which is present in the human heart, and allows the spread of the infection at a cellular level. The second one involves viral replication and dissemination in the bloodstream from Day 7 to up to one month after the initiation of the symptoms, leading to cytokine storm syndrome and direct myopericardial lesions caused by the infiltration of inflammatory cells [27]. Colchicine is a well-established and safe treatment for pericarditis [28] and appears to be the treatment of choice in COVID-19 cases due to its action on NLRP3 inflammasomes and cytokine release.

## Conclusions

This case is of particular interest since, to the best of our knowledge, it represents one of the first cases that documents encephalitis and myopericarditis as simultaneous complications of COVID-19 infection and highlights the complexity and diversity of clinical presentations associated with COVID-19. These patients may present with a wide range of symptoms, affecting multiple organs and systems, while the underlying mechanisms of such presentations can be elusive. The collection of extensive data about this disease is crucial for establishing diagnostic criteria and determining the most suitable treatment options. Central nervous system invasion might lead to a severe disability if not treated properly, while pericardial effusion induced by COVID-19 inflammatory syndrome could lead to cardiac tamponade. Early diagnosis and aggressive treatment seem to be the most crucial measures for these patients.
